# Pharmacists as youth-friendly service providers: documenting condom and emergency contraception dispensing in Kenya

**DOI:** 10.1007/s00038-020-01348-9

**Published:** 2020-05-22

**Authors:** Lianne Gonsalves, Kaspar Wyss, Peter Gichangi, Adriane Martin Hilber

**Affiliations:** 1 Department of Sexual and Reproductive Health and Research, including UNDP/UNFPA/UNICEF/WHO/World Bank Special Programme of Research, Development and Research Training in Human Reproduction (HRP), World Health Organization, Geneva, Switzerland; 2 Swiss Tropical and Public Health Institute (Swiss TPH), Basel, Switzerland; 3 University of Basel, Basel, Switzerland; 4 International Centre for Reproductive Health Kenya, Mombasa, Kenya; 5 Department of Human Anatomy, University of Nairobi, Nairobi, Kenya; 6 Ghent University, Ghent, Belgium

**Keywords:** Youth, Pharmacy, Family planning, Contraception, Sexual and reproductive health, Low-income country

## Abstract

**Objectives:**

This Kenya-based study ascertained whether pharmacies were an untapped source of ‘youth-friendly’ health services by determining (1) whether young people (aged 18–24) could successfully obtain condoms and emergency contraception (ECP); (2) whether contraceptives were dispensed according to national guidelines; and (3) how young people felt about obtaining ECP and condoms from pharmacy personnel.

**Methods:**

This study used several methods to capture and cross-check purchasing experiences as reported by young people with those of dispensing pharmacy personnel. These included: focus group discussions; in-depth interviews; key informant interviews; and mystery shoppers.

**Results:**

When in stock, young people were successfully able to obtain ECP and condoms from pharmacies. Counselling was sporadic: when it happened, it was not always accurate. Despite a lack of counselling, young people reported being satisfied with the quick, transactional interaction with pharmacy personnel.

**Conclusions:**

The brief, transactional interactions between pharmacy personnel and young clients appear to be ‘youth-friendly enough’. While there is room to strengthen the services provided (improving both accuracy and scope), this should be done in a manner that does not fundamentally alter the current interaction.

## Introduction

There are over 1.2 billion young people between the ages of 15 and 24 in the world today, 86% of whom live in low- and middle-income countries (United Nations Secretariat 2015). Young people face special vulnerabilities related to their sexual and reproductive health (SRH) and wellbeing (Patton et al. 2009; World Health Organization 2018a). They also struggle to obtain and use SRH information and services and family planning commodities from health facilities and health care providers (Chandra-Mouli et al. 2014). As a result, in many regions, young women wanting to avoid pregnancy can be up to twice as likely as adult women to have an unmet need for modern contraception (Guttmacher Institute 2010). Data from 61 low- and middle-income countries (LMICs) estimate that 33 million women aged 15–24 have an unmet need for contraception (MacQuarrie 2014).

In response, many countries have introduced ‘youth-friendly health services’ aimed at improving care for young clients by ensuring quality services meet established criteria around equitability, accessibility, acceptability, appropriateness, and effectiveness (World Health Organization 2012). However, challenges to effectively designing, implementing or scaling up these services mean that, in practice, facility-based youth-friendly health services may be unavailable or underutilized by their target population (Chandra-Mouli et al. 2014, 2015; High-Impact Practices in Family Planning (HIPs) 2015). Additionally, public sector facilities may not always have short-acting forms of contraception (particularly emergency contraception) available (Riley et al. 2018).

For youth unwilling or unable to go to a health facility, pharmacy provision of contraception—that is, over-the-counter (openly accessible at a pharmacy), or behind-the-counter (dispensing contingent on evaluation from a pharmacist) provision of contraceptives—can help to overcome barriers to access for young people (Chandra-Mouli et al. 2014; World Health Organization 2017). Studies from France, Australia, Canada, and the UK demonstrate that improved access to emergency contraception (ECP) through pharmacies results in high utilization among youth (Hobbs et al. 2011a, b; Marston et al. 2005; Moreau et al. 2006a, b; Soon et al. 2005). Additional studies have assessed how pharmacy access is operationalized (pharmacist personnel’s feelings about dispensing, young people’s actual ability to purchase contraception) (Gonsalves and Hindin 2017). Data from Kenya, Nigeria and Ethiopia also suggest that young people and unmarried people access emergency contraception from pharmacies (Corroon et al. 2016; Gold 2011); however, there is a general paucity of documentation from LMICs on what this access looks like in practice (Gonsalves and Hindin 2017).

In Kenya, young people between the ages of 15 and 24 constitute one-fifth of the total population (Kenya National Bureau of Statistics 2010). The 2014 Kenya Demographic and Health Survey found that short-acting modern contraceptive methods—condoms, pills and injectables, particularly—were popular among both married and unmarried sexually active young women aged 15–24 (Kenya National Bureau of Statistics et al. 2015). However, it also noted that among currently married young women, 23.0% of 15–19 year-olds and 18.9% of 20–24 year-olds still have an unmet need for family planning. Among sexually active unmarried women, 49.9% of 15–19 year-olds and 30.7% of 20–24 year-olds are not currently using any contraceptive.

Over the last twenty years, Kenya’s government has increasingly prioritized SRH programming for this age group, with an Adolescent Reproductive Health Policy (2003) which aimed to double the use of modern contraceptives among 15–24 year-olds by 2015 (Ministry of Planning and National Development/Kenya 2003) and the updated 2015 Adolescent Sexual and Reproductive Health Strategy aspiring to have 75% of 15–24 year-olds using a condom at sexual debut by 2025 (Ministry of Health/Kenya 2015). Additionally, Kenya’s National Family Planning Guidelines for Service Providers expressly supported provision of contraceptive services for adolescents and youth (Ministry of Health/Kenya 2018).

Importantly, these Family Planning Guidelines also authorize pharmacies to dispense various forms of contraception, including emergency contraception and male condoms (Ministry of Health/Kenya 2018). These can be accessed for free in Kenyan public facilities (Keesara et al. 2015) and are available for purchase from private pharmacies (sometimes locally referred to as “chemists”). Specifically, for emergency contraception (ECP), the guidelines authorize two available ECP types for pharmacies (Ministry of Health/Kenya 2018): combined oral contraceptives (the Yuzpe method), and dedicated progestin-only ECPs. The first dose for all available methods should be taken as soon as possible after unprotected sex, up to 120 h (World Health Organization 2018b). Anyone dispensing ECP is expected to explain how it works, and possible side effects, inform users of other available family planning methods, counsel on STIs or HIV/AIDS risks and testing, and refer for any needed SRH services. In dispensing condoms, the guidelines indicate that clients should be advised on proper use and disposal.

Against a backdrop of continued unmet need for contraception and supportive national policies for pharmacy provision of contraception, this study aimed to determine whether pharmacies in Coastal Kenya might be an untapped potential source of youth-friendly modern contraceptive sources. Specifically, we aimed to answer three questions: (1) can youth (aged 18–24) successfully obtain emergency contraception and condoms from pharmacies; (2) are these contraceptives dispensed according to Kenya’s National Family Planning Guidelines; and (3) how do youth purchasing ECP or condoms act and react to the experience?

## Methods

This analysis was part of a broader, mixed methods study aiming to understand what drives young people in Kwale County in need of any contraception to obtain it from pharmacies. Emergency contraception and condoms are the focus of this analysis given their over-the-counter availability globally (International Consortium for Emergency Contraception 2013), their popularity among young people in the study area (data unpublished), their connection to a single sex act, and the fact that they are self-administered (rather than requiring intervention from a trained provider).

Kwale County is one of six counties in Kenya’s former Coast province, and young people aged 15–24 are estimated to comprise 19% of the County’s total population (Kwale County Economic Planning Division 2017). This study took place in the peri-urban towns of Kwale and Ukunda, as well as the stretch of highway connecting the two. Data were collected between November 2017 and March 2018. Young data collectors, speaking both English and Swahili, were recruited from the study area and from nearby Mombasa and specifically trained for this study. As captured in Table 1, this study used several methods to capture and cross-check experiences as reported by young people aged 18–24 who might purchase contraception from pharmacies, and pharmacy personnel tasked with dispensing contraception.

**Table 1:** Description of study methods (Kenya, 2017–2018)

	Participant inclusion criteria	Topics addressed	*N*	Youth perspective	Dispenser perspective
Focus group discussions	Age 18–24	Locations in the community where young people obtain contraception and why The type of young person who would rely on each given location	6 (58 participants)	x	
In-depth interviews	Age 18–24 Recently purchased contraception from pharmacy	Detailing personal experience buying contraception from a pharmacy Reasons for choosing to buy contraception at a pharmacy	18	x	
Key Informant Interviews	Age 18 + Currently works (in any capacity) at pharmacy OR Pharmacy-related stakeholder (Ministry of Health; professional association; non-governmental organization)	General personal/pharmacy policies for dispensing contraception Detailing personal experience dispensing contraception to young people Feelings about dispensing contraception to young people	19 (pharmacy personnel) 6 (stakeholders)		x
Mystery shopper	*Trained data collectors (aged 18–24) served as mystery clients*	Documenting purchase-related interactions with pharmacy personnel Objective assessment of the interaction	142 visits	x	x

Six Focus Group Discussions (FGDs) with young people aged 18–24 (three FGDs with young men, three with young women) were conducted with 8–10 participants each. We also conducted 18 in-depth interviews with young people who had recently purchased some form of contraception from a pharmacy. Recent-purchasers were recruited through local pharmacies: after a young person had purchased the contraception, participating pharmacists were instructed to provide a leaflet with information about the study to the client. Additionally, one young data collector spent three evenings stationed outside of two pharmacies with high client traffic and recruited young people who had just purchased contraception.

Data collectors mapped all the pharmacies in the study area. Pharmacies (whether open or closed at the time of the visit) were mapped via digital form with an embedded geolocator. A total of 60 pharmacies were enumerated. Data collectors conducted interviews of pharmacy personnel in a random sample of the enumerated pharmacies. A total of 19 interviews were conducted, and data collectors interviewed whomever they found dispensing medicines behind the counter, regardless of their qualifications or formal role. An additional six key-informant interviews were conducted with Kwale County and Federal-level Ministry of Health stakeholders, as well as representatives from the Pharmaceutical Society of Kenya (the professional association for practicing pharmacists) and non-governmental organizations working on contraception access.

During the mapping process, 50 of the 60 pharmacies also consented to be visited by mystery shoppers. Two of the 50 participating pharmacies were permanently closed when data collection began, leaving 48 pharmacies to be visited by each shopper. Three mystery shoppers (two male, one female) visited these pharmacies with fictitious personas reflecting common situations in which a young Kenyan would need contraception. The personas (further detailed in Table 2) were developed jointly with youth from the area and informed by preliminary findings from the qualitative data collection described above. Mystery shoppers were provided a set amount of money with which to purchase their assigned contraceptive. All mystery shoppers were instructed to answer questions put to them by pharmacy attendants; however, their self-initiated interactions were limited to asking for their specific contraception. Each mystery shopper piloted their persona in two pharmacies outside of the study area prior to beginning data collection.

**Table 2:** Description of mystery shopper personas (Kenya, 2018)

Mystery shopper (MS)	Persona description *Characters were developed to reflect common scenarios which might send a young person to a pharmacy for ECP or condom. Additionally, they reflect typical backstories, concerns, and knowledge that young people purchasers were perceived to have*
MS 1 emergency contraception (female)	Student, 22 years old. Had sex with her boyfriend two days ago. They used a condom, but it burst. This is her first time using ECP. She does not know her boyfriend’s HIV status but is only worried about pregnancy, not HIV/STIs. Has 100 Kenyan shillings to purchase ECP
MS 2 emergency contraception (male)	Student, 21 years old. Has a girlfriend but he doesn’t use a condom during sex because ‘he trusts her’—they use ECP instead. He is purchasing ECP on his girlfriend’s behalf and does not know the instructions for use. Has 100 Kenyan shillings to purchase ECP
MS 3 condom (male)	Secondary school graduate, 19 years old. Single, known as a ‘hit and run’ (someone who has sex with many women but does not date them). He is going to a party in the evening, and wants to purchase condoms in advance. Knows about HIV/STIs but more worried about pregnancy. Has 50 Kenyan shillings to purchase condoms

### Data collection

Semi-structured interview guides were developed and used for focus group discussions, key informant interviews, and in-depth interviews. After obtaining informed consent from participants, all FGDs and interviews were audio recorded. Qualitative data collection were in Swahili, English, or a mix of the two, depending on the participant’s preference. The time and location of interviews were set based on participants’ availability and preference: in the case of pharmacy personnel and stakeholders, this was often the participant’s place of work. Mystery shoppers were provided with short, semi-structured digital forms to complete on mobile phones. The forms were adapted from a previous family planning mystery shopper study (Chin-Quee et al. 2006) and included questions about the pharmacy, its staff, and the purchase-related interaction. Mystery shoppers were instructed to complete the form immediately after each visit.

This study received ethics approval from the Ethikkommission Nordwest- und Zentralschweiz (EKNZ) (Req-2017-00389) in Basel, Switzerland, as well as the University of Nairobi/Kenyatta National Hospital in Nairobi, Kenya.

### Analysis

All qualitative data were transcribed and translated (if necessary) into English. Data collection and analysis were informed by an iterative approach, allowing for adapting and modifying of question guides and checklists as data collection evolved. The qualitative data were analyzed through thematic analysis, using inductive and deductive coding (the latter of which was informed by a research objective to understand dispensing practice) on an initial cross-section of transcripts to develop a coding framework. Qualitative analyses were conducted in Atlas.ti Version 8. Mystery shopper data were analyzed in Stata version 14, with analysis consisting of descriptive statistics.

## Results

### Young people successfully obtain condoms and ECP from pharmacies

Focus Group Discussion and interviews indicated that young people could readily access both emergency contraception and condoms from pharmacies in the study area. The mystery shopper exercise confirmed this. As seen in Table 3, emergency contraceptive pills and condoms—when in stock—were always dispensed to the mystery shoppers. The female ECP shopper successfully purchased emergency contraception in every pharmacy she visited. The male ECP shopper was nearly as successful, being denied ECP in only one pharmacy due to its being out of stock. The condom shopper was able to purchase a pack of three condoms at 33 pharmacies and left empty-handed if condoms were out of stock (in six pharmacies) or if they were too expensive (in five pharmacies). One additional pharmacy did not sell condoms, and two more pharmacies were closed when visited.

**Table 3:** Number of pharmacies visited, number of successful purchases, and ‘additional interactions’ (above and beyond the purchasing itself) between pharmacy personnel and mystery shopper, by mystery shopper (Kenya, 2018)

	Pharmacies visited (*N* = 48)	Successfully purchased family planning	Any additional interaction*	Additional interaction details
Mystery shopper (MS)		*n* (%)	*n* (%)**	(*n*)***
MS 1 emergency contraception (female)	47	47 (100)	8 (17.0)	Timing of unprotected sex (1) MS age and student status (1) Brand description/recommendation (6) Instructions on use (4)
MS 2 emergency contraception (male)	48	47(98)	4 (8.5)	Timing of unprotected sex (1) Whether MS knew how to use emergency contraception (1) Brand description/ recommendation (1) Instructions on use (2)
MS 3 condom (male)	47	33 (70)	4 (12.1)	Brand description/recommendation (4)
Total	142	127 (89)	16 (12.6)	

^*^Additional interactions were described as any interaction beyond the purchase itself, and were broadly grouped into three categories: (1) assessment of need/prior use; (2) personal questions; (3) assistance selecting contraception (including instructions on use)

^**^Percentages are presented as a proportion of any additional interaction in pharmacies where contraception was successfully purchased

^***^Number of pharmacies where additional interaction took place—note, multiple interactions may have taken place in the same pharmacy

### Condoms and ECP are not consistently dispensed with counselling

Most interview participants reported that young clients only received counseling if they requested it. Otherwise, they were just given the contraception.

“We don’t bother unless they ask us, ‘Oh, I don’t know how to use this.’ That is when we can explain but if they don’t, we just dispense and they are gone.”—Pharmacy attendant (no formal training) (Pharmacy A2).

“when you ask questions, they [pharmacy personnel] explain to you and they advise you…unless you do not want advice. But when you ask any question on family planning, they answer you.”—Youth interview 10, female, has purchased ECP and condoms.

Among pharmacy personnel who admitted that they dispensed contraception without counselling, some did so under an assumption that a client asking for a specific product already knew how to use it.

“now they will want for example, the emergency pill…sometimes, there is that kind of atmosphere with the person, so you dispense it because they kind of know what they want, they have asked their friends.”—Pharmaceutical technologist (Pharmacy F2).

More often, however, pharmacy personnel indicated that they could see that a young client did not want questions, advice, or prying.

“A girl coming to buy e-pills…just telling them how to use that drug is a challenge, she will pick it up and right away put it in her bag and does not want any explanation about it from you.”—Pharmacy personnel (training not specified), (Pharmacy B1).

Several pharmacy personnel insisted that young clients received some counseling when purchasing emergency contraception. However, the counselling described consisted of ECP counselling and eligibility screening based on inaccurate information. For example, participants thought ECP was usable only up to 72 h following unprotected sex, rather than 120 h. This may be a result of commercial brands of ECP in Kenya still being labeled for use up to 72 h, and pharmacy personnel reading this labeling.

[re-enacting a purchase with a fellow pharmacy attendant] “When you have had sex, let not 72 h pass… after 72 h this drug will not be of help but before that it is okay. And it is important to know your safe days…so, if you are sure you are unsafe I will sell to you the drug.”—Pharmacist (Pharmacy C10).

Reported counselling also included warnings that ‘overuse’ of ECP could result in health problems (another unfounded concern).

“I tell her that these [emergency] pills are good and bad as well, because if you use them consecutively for six months, they might cause a growth in your stomach. Other times, it is said they can bring problems when you are trying to have a baby.”—Pharmaceutical technologist (Pharmacy C9).

In practice, the mystery shopper exercise (detailed in Table 3) confirmed a lack of engagement between pharmacy personnel and young people, including around issues which would ensure quality service provision. For example, each ECP shopper was asked about the timing of unprotected sex (critical for determining eligibility) in only one pharmacy. ECP shoppers received instructions on how to take the pills—also an expected interaction—in four pharmacies for the female ECP shopper, and twice for the male ECP shopper.

The lack of engagement also extended to unnecessary questioning. ECP shoppers were asked very few personal questions regarding age, relationship status, and/or whether they were students (none of which are relevant to determining their ability to access ECP). In one pharmacy, for example, the female ECP shopper reported receiving a ‘little lecture’ from a ‘shocked’ pharmacy attendant. The male ECP shopper also reported that one pharmacist indicated that he should ‘give money to his girlfriend so she could come herself’; however, he was still able to purchase the ECP. Finally, in a handful of interactions, pharmacy personnel shared their personal views on ECP brands, including perceived differences in cost, effectiveness, and quality, as well as personal favorites.

Engagement between pharmacy personnel and the condom mystery shopper was even more hands-off: the *only* non-purchase-related interaction for this shopper was pharmacy personnel in four pharmacies sharing their personal preference for condom brands. It is worth emphasizing that any interactions documented by the three mystery shoppers were the exception rather than the norm: in 111 out of a total 127 mystery shopper-pharmacist interactions, shoppers entered, requested their contraceptive, received it, paid for it, and left. There was no additional interaction with the person behind the counter.

### Young people are pleasantly surprised by polite, transactional purchasing experiences

Both pharmacy personnel and youth participants reported that young purchasers initially approached pharmacies with trepidation. Young purchasers worried about the pharmacy attendant being older and/or of the opposite sex; what they would be required to disclose about their personal lives; and what lecture they might have to endure as a result.

“I was relieved, because I thought he was going to judge me or lecture me but it was a relief knowing it was easy to purchase. I thought he was going to ask if I had an ID and [say] that I was still young and what in the world was I going to do with them because they were meant for adults…but he did not.”—Youth interview 16, male, has purchased ECP and condoms.

As a result, both groups reported that young people sometimes sent friends or partners to purchase on their behalf. If they went themselves, participants reported the young person waiting to purchase contraceptives until the pharmacy was empty.

[Describing a young person purchasing a condom] “It is something that needs a lot of courage in a young person…some send others instead. Some wait until no others customer is present at the chemist. They check first [and then] they approach. Even if he came first, others get served first and then go so that he may get his turn. He’ll say ‘I’m in no rush, serve them.’” -Trained pharmacist or pharmaceutical technologist (not specified), (Pharmacy D5).

Once in the pharmacy, young people had strategies to keep their contraceptive purchase discreet.

“Say for example, a young man comes in and he wants ‘Trust’ [a popular condom brand]. He will gesture, pointing. ‘Can I have that?’ He is pointing, so, if you are not keen you might not know what the young man wants. Alternatively, they will use coded language, which luckily enough, we happen to know.”—Pharmacist (Pharmacy I1).

Specific codewords identified included ‘lifesavers’ or ‘our tablets’ for emergency contraception and ‘balloon’ or ‘socks’ for condoms. The use of codes and gestures (pointing, or miming what was needed) stemmed from discomfort or embarrassment with requesting these contraceptive methods out loud or being overheard by other customers. This same fear contributed to young participants’ appreciation of brisk, transactional interactions with pharmacy personnel, devoid of counselling.

“He did not give me any advice, because when I went there, I did not want any stories. I wanted him to give me what I wanted [and] then I leave. I did not want to create a conversation because I feared other people could walk in and find me purchasing”—Youth interview 17, male, has purchased condoms.

After the purchase was complete, young purchasers indicated they left in a hurry, with a lingering worry that a pharmacy attendant would think poorly of them. However, overall, despite needing to “gather courage” to enter the shop, every young purchaser interviewed described polite, transactional experiences purchasing condoms and emergency contraception. In fact, more than one third of the young purchasers interviewed reported that they had purchased contraception from pharmacies more than once.

“The interaction is very fast, because you want to buy and leave. He served me well, because he took it that I was a young person who just wants to avoid the pregnancy and protect herself.”—Youth interview 10, female, has purchased ECP and condoms.

## Discussion

Our findings indicated that a young Kenyan, aged 18–24 who wished to purchase either condoms or emergency contraception from a pharmacy could expect to successfully do so via a brisk, non-intrusive interaction with pharmacy personnel. This meant that young ECP purchasers rarely received eligibility screening or instruction on use. However, it also meant that pharmacy personnel rarely subjected young purchasers to unnecessary, values-based questioning related to marital/relationship status, age, or the appropriateness of engaging in sex. With some exceptions, counselling was only provided when the client asked for it [in line with previous findings from a mystery shopper exercise in Kenya (Liambila et al. 2010)], out of an assumption that the young purchaser prioritized speed and discretion over information. When this happened, young clients were satisfied with the information they received and way it was conveyed. Interactions with pharmacy personnel that were neutral at worst and warm at best left them pleasantly surprised and more comfortable returning to the pharmacy in the future. The pharmacy purchase experience in its current form, for these contraception types, was ‘youth-friendly enough’.

Recognition that young people should be able to access quality health services, including SRH services, in a ‘youth-friendly’ environment has resulted in a focus on improved public SRH services (Chandra-Mouli et al. 2015; UNAIDS Inter-agency Task Team on Young People 2006). The role of the private sector, specifically pharmacies, as an important source of contraception has been less clear. WHO’s 2001 consensus statement from a Global Consultation on Adolescent Friendly Health Services acknowledged that adolescents used pharmacies and that these anonymous, rapid, and numerous contraception sources warranted further study (World Health Organization 2002). Nearly 15 years later, a systematic review found that assessments of most out-of-facility interventions (including pharmacies) as effective providers of ASRH services were still lacking (Denno et al. 2015). This study is contributing to the body of knowledge by documenting the role of pharmacies as a source of youth-friendly services.

Clients vote with their feet and in Kenya, as in many countries, the popularity of pharmacies for ECP is clear (Benevides et al. 2014; Kenya National Bureau of Statistics et al. 2015; Liambila et al. 2013). Progestin-only emergency contraception in particular has been extensively studied and deemed safe (World Health Organization 2018b) and appropriate for over-the-counter sale (International Consortium for Emergency Contraception 2013), as condoms are. This makes engagement with a pharmacist (per global evidence), ideal but not necessary. That said, these current interactions are still a missed opportunity to discuss SRH more broadly and bridge interested young people to a more regular form of contraception. The challenge, therefore, is to improve pharmacy personnel-youth interactions around ECP and condom dispensing without fundamentally modifying an interaction which appears to be youth-friendly enough for young people (Liambila et al. 2013).

Creative ways to unobtrusively link young customers with additional information or services (Liambila et al. 2013), such as a digital health campaign(L’Engle et al. 2016) or an easy-to-read leaflet in a bag (Both and Samuel 2014), can provide young people with instructions on use and further contraception referrals they may be open to, at a moment of their choosing. Youth-targeted information and education (IEC) materials displayed in pharmacies (Both and Samuel 2014) can provide basic information, address common concerns, and encourage young people to speak up and discuss their purchase with pharmacy personnel. IEC materials also become a resource for pharmacy personnel themselves.

This study demonstrated that there appear to be lingering and pervasive misconceptions among trained and untrained pharmacy personnel about ECP’s effectiveness beyond 72 h as well as its safety. Despite clear consensus about ECP’s safety and window of use (World Health Organization 2010), the misinformation about ECP’s safety is not limited to pharmacists. A previous review demonstrated that a variety of providers in various LMIC settings shared concerns about ECP possibly causing congenital abnormalities, infertility, even cancer (Dawson et al. 2015). There is also apparent disconnect between what national (and international) guidelines say for ECP’s window of use (120 h) and what registered drugs on the market advertise (72 h). This discrepancy likely contributes to continued confusion among pharmacy personnel.

Collaboration between professional associations, pharmaceutical training institutions, Ministry of Health, and even ECP retailers, can provide pharmacy personnel with improved pre-service training as well as in-service refreshers (Benevides et al. 2014; Liambila et al. 2013) and resources which dispel myths and reinforce important messages around ECP and contraception more broadly. Any initiatives to strengthen pharmacy services will have to consider opportunities and challenges created by their dual role as health service provider and for-profit business. For example, at present, pharmacists will not likely deny a young person contraception; at the same time, they have no particular incentive to provide additional counselling.

This study and analysis had certain limitations. The goal of the mystery shopper exercise was to observe dispensing practices of pharmacy personnel, without any prompting from a client. As such, this exercise could not probe pharmacy personnel on their beliefs about emergency contraception or systematically test the accuracy of the ECP-related counseling among pharmacy personnel: further research might explore these. Additionally, during the early rounds of interviews with pharmacists, we discovered barrier methods like condom and the post-facto ECP were not always considered ‘family planning’ by this group. This created a challenge during data analysis in determining whether ECP was included when pharmacy personnel shared their thoughts about contraception in general: as a result, we only included data that made explicit reference to ECP or condoms. Also, as we visited all pharmacies to obtain consent for a mystery shopper visit prior to the exercise, there is a risk of observation bias—that is, pharmacies modifying their normal treatment of young people knowing that one customer might be a mystery shopper. That said, our mystery shopper exercise only began about two months after the initial visit. Finally, social desirability bias likely caused participants to overstate the level of pharmacist-youth engagement for the benefit of our data collectors. This would explain the discrepancies between qualitative participants reporting more engagement than our mystery shoppers observed in practice, and reinforces the benefit and importance of triangulation across different methods, especially in research which explores culturally sensitive topics.

### Conclusion

As a preferred source of condoms and ECP for young people, this study demonstrated that pharmacies in Coastal Kenya currently dispense ECP and condoms in a manner responsive to young customers’ self-reported needs. In Kenya, pharmacies have an essential role to play in youth SRH service provision, which could be capitalized upon under the country’s broader health agenda. In 2017, Kenya’s president unveiled his ‘Big Four’ development agenda, which included universal health coverage as one pillar (Otinga 2018). Under this umbrella, there has been interest to link with private sector healthcare providers in order to improve the reach of health services beyond what the public sector can offer (The Star 2018).

If pharmacy-based care were considered under this initiative, it would necessitate close collaboration with pharmacy stakeholders, public health service providers, and especially young people to ensure quality of care, and continued youth-friendliness. However, for an otherwise healthy young person, a visit with the neighborhood pharmacist may be their only health system engagement for their SRH: exploring improvements and expansions of SRH services in pharmacies is a worthwhile undertaking.
